# Understanding the Affective and Mental Health Outcomes of Meditation Interventions: The Role of Individual Differences in Self-compassion

**DOI:** 10.1007/s12671-026-02803-z

**Published:** 2026-04-08

**Authors:** Manuella B. Kury, Barbara L. Fredrickson, Patty Van Cappellen, Brian P. Don

**Affiliations:** 1 University of Auckland, Auckland, New Zealand; 2 University of North Carolina at Chapel Hill, Chapel Hill, United States; 3 Duke University, Durham, United States; 4 The University of Texas at Austin, Austin, United States

**Keywords:** Mindfulness meditation, Loving-kindness meditation, Self-compassion, Emotion, Depression, Guilt and shame

## Abstract

**Objectives:**

Extensive research documents that mindfulness meditation (MM) and loving-kindness meditation (LKM) have benefits for emotional, social, and mental health outcomes. Despite this work documenting the benefits of meditation practice, research also demonstrates that meditation does not always have equivalent effects for all people. So, who is most likely to benefit from meditation practice? In this study, we proposed that individual differences in baseline levels of *self-compassion* may moderate the association between meditation training and affective, social, and mental health outcomes across time.

**Method:**

To test this proposition, we used data from a randomized intervention study (collected from 2013 to 2015) where 217 adults received 6 weeks of training in either MM or LKM, and reported their emotions, feelings of social connectedness, and depressive symptoms.

**Results:**

Consistent with our pre-registered hypotheses, results from multilevel analyses demonstrated that participants who were lower in self-compassion prior to the intervention tended to experience greater affective and mental health benefits across the course of the intervention period. Moderated mediation analyses demonstrated that increases in self-compassion from pre- to post-intervention mediated the relationship between training in meditation and lower depression across time, but only for those with lower baseline self-compassion levels.

**Conclusions:**

These findings spotlight the role of individual differences in self-compassion in contributing to people’s response to meditation interventions, suggesting that people low in self-compassion may stand to benefit the most from meditation training.

**Pre-registration:**

This study was pre-registered and the pre-registration can be viewed on https://aspredicted.org/48ik9.pdf.

**Supplementary Information:**

The online version contains supplementary material available at 10.1007/s12671-026-02803-z.

Extensive research documents that mindfulness meditation (MM) and loving-kindness meditation (LKM) tend to be effective in contributing to better mental health, such as improved symptoms of depression and anxiety, but also in increasing positive emotions and decreasing negative emotions (Fredrickson et al., 2017; Goldberg et al., 2022; Goyal et al., 2014; Lv et al. 2024; Lindsay et al., 2018; Reangsing et al., 2021; Zeng et al., 2015; Zheng et al., 2023). Despite this, although MM and LKM meditation are promising strategies to promote well-being, research on MM and LKM is not always consistent: while many studies document the benefits of meditation interventions, the effect size of any given meditation intervention can vary depending on the population, outcome, or specific problem of interest (for instance, see Goldberg et al., 2022 for a quantitative review of the mindfulness-based intervention literature). Given the variability in effect sizes in the existing literature, it is important to understand *why* effect sizes tend to vary between meditation training studies.

In this research, we proposed one critical yet heretofore underexamined factor that may partially explain the variability in effect sizes in prior meditation training research: the role of *individual differences in self-compassion* (Neff, 2003, 2023). Specifically, we proposed that MM and LKM would be particularly beneficial to individuals who are *low* in self-compassion because these individuals theoretically have the most to gain from training in MM and LKM. To examine this hypothesis, we used data from a randomized intervention study of 217 adults who were trained in MM and LKM across the course of 6 weeks, while reporting on their emotions, social connectedness, and mental health outcomes.

MM, which originates in Buddhist teachings and practices (Kabat-Zinn, 2011), involves training one’s attention to focus on the present moment in an open, nonjudgmental manner (Creswell, 2017; Lindsay & Creswell, 2017). In MM, the focus on the present moment can be directed at many targets, including attention to bodily sensations, emotional experiences, thoughts and mental images, and perceptual experiences, such as sounds in the present environment (Creswell, 2017). In addition to focusing their attention on present moment experiences, in MM practitioners are trained to be *accepting* of present moment experiences. That is, they are taught to approach their experiences in an open, nonjudgmental manner, regardless of whether these experiences are enjoyable, unpleasant, or simply neutral.

Extensive research demonstrates that MM tends to beneficially contribute to enhanced affective outcomes, mental well-being, and social connectedness. For instance, Fredrickson et al. (2017) found that training in MM was associated with enhanced positive emotions (see also Lindsay et al., 2018), and other studies have demonstrated that MM is associated with decreases in negative emotions (e.g., Keng et al., 2011). Many studies have also found MM interventions to be effective in improving symptoms of anxiety and depression (Goldberg et al., 2022; Grogan, 2025; Reangsing et al., 2021).

LKM originates in the Buddhist tradition and involves cultivating a state of compassion and kindness towards others and oneself (Hofmann et al., 2011; Salzberg, 1995). LKM practice consists of directing kind and warmhearted thoughts to specific targets (Zeng et al., 2015), typically towards oneself, loved ones, neutral targets, strangers, individuals that the person has difficulties with, and ultimately to all beings (Galante et al., 2014). Although MM and LKM have many similarities, one of the primary differences between the two is that LKM encourages individuals to actively cultivate compassion towards the self and others, even when that is not what the individual is already experiencing in the present moment.

Extensive research has linked LKM to beneficial affective experiences, mental health outcomes, and social outcomes. For instance, recent quantitative reviews of the literature have documented that LKM is linked with increases in positive emotions, fewer depressive symptoms, and lower levels of anxiety (Lv et al., 2020; Petrovic et al., 2024; Zeng et al., 2015; Zheng et al., 2023; Zheng et al., 2025). Additionally, research demonstrates that LKM is related to enhanced feelings of social connectedness (Hutcherson et al., 2008; Seppala et al., 2014).

Although the existing literature shows that MM and LKM promote beneficial outcomes, many questions remain unanswered. While extensive research demonstrates that MM and LKM promote beneficial outcomes like increased positive emotions, better mental well-being, and reduced stress, it is important to note that meditation interventions often have varying effect sizes depending on the population, outcome of interest, or type of meditation intervention (Goldberg et al., 2022; Goyal et al., 2014; Lv et al. 2020; Zeng et al., 2015; Zheng et al., 2023). Indeed, according to a review of the literature by Goldberg et al. (2022), mindfulness-based interventions appear to have transdiagnostic relevance, *but not in all populations or for all issues*. The goal of this research is to illuminate an individual difference factor (self-compassion) that may render meditation interventions more or less effective depending on the fit between the intervention and the person (Lyubomirsky & Layous, 2013).

As reviewed above, extensive evidence demonstrates that MM and LKM have beneficial effects, yet variability in the effect of meditation on key outcomes is commonplace. Why is this the case? While there are indeed many possible reasons, in this research we proposed that *individual differences* (in this case, individual differences in self-compassion) play a part in how people respond to MM and LKM. That is, consistent with a small but growing literature examining individual differences in response to meditation (Barczak-Scarboro et al., 2021; Buric et al., 2022; West et al., 2022), we proposed that these meditation practices may be especially helpful to certain individuals in terms of their emotional, mental health, and social outcomes. The fact that many prior studies have examined the overall influence of MM and LKM while not examining or accounting for these individual differences could obscure precisely *who* these meditations are effective for, thus at least partially explaining the varying effect sizes documented in prior research in this area. We note that while some researchers have discussed the role of individual differences in terms of the *adverse* outcomes of meditation (e.g., Dobkin et al., 2012), our goal in this work is to illuminate the possibility that meditation training may have particular affective, social, and mental health *benefits* for some people, whereas it may be less beneficial for others.

Some prior research suggests that individual differences may be critical in determining the beneficial affective outcomes of MM and LKM. For instance, in a study where participants were assigned to receive training in MM and LKM in a 6-week intervention, West et al. (2022) found that individuals with higher baseline attachment anxiety experienced greater increases in positive emotions and reductions in negative emotions, compared to those with lower attachment anxiety. Other work has demonstrated that (a) individuals with low levels of resilience prior to meditation training tend to experience the greatest benefits (Barczak-Scarboro et al., 2021), (b) baseline levels of trait mindfulness predict key outcomes following mindfulness interventions (Barcaccia et al., 2024), and (c) baseline fear of self-compassion predicts the efficacy of self-compassion interventions (Liu et al., 2023). Building on these prior studies, in the current work, we proposed that self-compassion is a key individual difference variable that may be associated with how people respond to both MM and LKM.

According to Neff (2003, 2023), self-compassion consists of three core elements: self-kindness, a sense of common humanity, and mindfulness. Self-kindness refers to an individual’s capacity to treat themselves with warmth, understanding, and non-judgement. A sense of common humanity is a person’s capacity to realize that all humans make mistakes. Finally, mindfulness in this context refers to the extent to which individuals tend to be aware and accepting of present moment experiences.

Extensive research has documented the connection between MM and LKM and self-compassion (Golden et al., 2021; Lv et al., 2024; Tal-Saban et al., 2024). For instance, numerous pre-post design studies and randomized control trials have demonstrated that those who practice MM and LKM tend to experience increases in self-compassion (Galantino et al., 2005; Krasner, 2009; Moore, 2008). Moreover, some studies have demonstrated that increased self-compassion mediates the link between these meditation practices and the beneficial outcomes they tend to produce (Baer et al., 2012; Kearney et al., 2013). Importantly, although research demonstrates MM and LKM are associated with increases in self-compassion (which can partly explain why these meditation practices are beneficial), no research has tested whether individual differences in baseline levels of self-compassion influence how effectively people respond to these meditations.

Based on prior theory and research, we proposed that individuals *lower* in baseline levels of self-compassion would be particularly likely to benefit from training in MM and LKM meditation, whereas those higher in baseline levels of self-compassion would be less likely to benefit. We made this proposition because MM and LKM tend to address the three central facets of self-compassion—self-kindness, common humanity, and trait mindfulness—from which people low in baseline levels of self-compassion are particularly likely to benefit. By contrast, people higher in baseline levels of self-compassion tend to already be high in self-kindness, common humanity, and trait mindfulness, meaning they have less room to benefit from the skills they are learning in MM and LKM.

With respect to the affective outcomes of meditation, we chose to focus on *self-conscious emotions*—specifically, daily (a) guilt and shame and (b) pride (Tracy & Robins, 2004). We focused on these self-conscious emotions because we theorized they would be particularly relevant to individual differences in self-compassion. Shame refers to global negative feelings about the self, whereas guilt refers to negative feelings about a specific action that the individual has engaged in (Tracy & Robins, 2004; Niedenthal et al., 1994). Self-compassion is negatively related to guilt and shame (Mosewich et al., 2011), and according to self-compassion theory, people who are low in self-compassion are particularly likely to experience these feelings due to their high levels of self-criticism (Neff, 2003). As they learn to be more kind and accepting towards themselves during MM and LKM training, we theorized that people with low levels of self-compassion would experience especially prominent decreases in guilt and shame. By contrast, we suspected that people with high levels of self-compassion would already tend to engage in self-acceptance and kindness, meaning MM and LKM may not lessen the incidence of these self-conscious negative emotions for these people.

Pride tends to refer to one’s recognition of their own accomplishments and efforts to reach a goal (Tracy & Robins, 2007). According to self-compassion theory, because of their less kind views towards themselves, those with lower levels of self-compassion tend to harbor harsh judgments towards themselves and ruminate on their flaws, whereas individuals who are higher in self-compassion will not. Indeed, individuals with greater levels of self-compassion tend to report lower fear of failure, lower perfectionism, and lower self-criticism in achievement domains (Stoeber et al., 2020). This evidence suggests that, for someone with low self-compassion, any minor mistake, failure to meet a goal, or perceived lack of competence may result in a threat to their sense of pride (e.g., Neff, 2023; 2005; Stoeber et al., 2020). After training in MM or LKM, we theorized that individuals who are low in self-compassion should become better able to treat themselves with kindness and care, and thereby recognize their own accomplishments and experience pride. By contrast, it is possible that people high in baseline self-compassion—who tend to have lower levels of perfectionism and fear of failure—would already tend to experience high levels of pride in everyday life prior to meditation training.

Baseline levels of self-compassion are likely to be related to social connectedness in response to meditation interventions. People low in self-compassion tend to believe that they are alone in their pain and fail to recognize that others face similar challenges and difficulties, thus decreasing their feelings of connection to others (Neff, 2003). In addition, people with low self-compassion tend to have more challenging relationships and struggle to respond constructively to conflict, which is likely to make them feel more alone (Neff & Beretvas, 2013). As such, MM and LKM may help people low in self-compassion (a) feel that their experiences are connected with others and (b) provide them with equilibrium in their ongoing relationships, both of which could help them feel less isolated.

In addition to the aforementioned outcomes, we examined the possibility that baseline levels of self-compassion would be associated with changes in depressive symptoms in response to meditation training. Depression is characterized by rumination, greater elaboration on negative information, and negative views of the self (Gotlib & Joormann, 2010). People with lower self-compassion tend to have higher symptoms of depression since they tend to ruminate about their problems and have a more critical view of themselves, as compared to those with higher self-compassion (Liu et al., 2022; MacBeth & Gumley, 2012; Neff, 2003). Because MM and LKM help people cultivate key self-compassion skills, it is likely that people with lower levels of self-compassion would be especially likely to report reductions in, and that increases in self-compassion would mediate these reductions in depression.

Given that there are many potential moderators to the efficacy of meditation practice (e.g., baseline psychopathology, motivation, etc.; Buric et al., 2022), why examine self-compassion specifically? We chose this specific moderator in part because of theoretical perspectives like the positive-activity fit model (Lybomirsky & Layous, 2013), which suggests that response to any specific intervention is dependent on both features of the person and the intervention. With respect to these meditation interventions, one of the core skills that both MM and LKM teach is self-compassion. When people repeat kindhearted messages in LKM, or learn to view their present-moment experiences with acceptance in MM, they are learning to cultivate self-compassion (Galantino et al., 2005; Krasner, 2009; Moore, 2008). As such, it is possible that when considering the person-activity fit of these meditation interventions, baseline self-compassion could be an integral factor in determining who tended to benefit from engaging in them.

The goal of this study was to examine whether individual differences in baseline levels of self-compassion moderate the link between training in MM and LKM and affective and mental health outcomes. We hypothesized that, as compared to those higher in self-compassion, those with lower self-compassion would experience better outcomes in response to meditation training, including a bigger decrease in daily self-conscious negative emotions (guilt and shame), as well as a bigger increase in daily pride and social connectedness. We predicted that these findings would emerge both across the course of the intervention and in response to meditation on a particular day. We also predicted that participants lower in self-compassion would experience a bigger decrease in depression from before to after the intervention, and that this differential reduction would be mediated by greater increases in self-compassion for those lower in baseline self-compassion.

To test these hypotheses, we drew upon an archival study of individuals with no previous meditation experience who were randomly assigned to receive 6 weeks of training in MM or LKM. Participants completed a baseline assessment of their self-compassion levels, as well as one after the end of the meditation intervention. They also completed daily assessments of their meditation frequency and emotions, and social connectedness throughout the course of the study, including before and after the meditation training. Additionally, they completed an assessment of depression before the meditation intervention and after it was over. Our hypotheses and data analyses were pre-registered, and the pre-registration can be viewed at the following link: https://aspredicted.org/48ik9.pdf. We note that, although we pre-registered the UCLA Loneliness Scale as an outcome, subsequent to the pre-registration, we realized this scale was not available in full at the laboratory sessions, meaning we were not able to conduct the analyses we outlined for this variable in the pre-registration. Social connectedness was assessed at the daily level, so we instead used this variable to mirror the daily guilt, shame, and pride analyses throughout the paper.

## Method

### Participants

This study included 217 participants from an archival study in which participants were randomly assigned to receive training in either MM or LKM, although the exact number of participants included in our subsequent analyses depended upon the questionnaires that participants completed. This data has been used in previous publications (Don et al., 2022; Fredrickson et al., 2017), and comes from a study funded by the National Institutes of Health. Participants were recruited from the community around a large university in the Southeast of the USA. The participants were between 34 and 64 years old and interested in healthy lifestyle changes. Participants were eligible for the study if they had no previous meditation experience, did not have a disability or chronic illness, had access to the internet at their home, and were fluent in written and spoken English. Participants were 48.97 years old on average (*SD* = 8.91). Regarding gender, 40.20% identified as men and 59.70% identified as women. In addition, 77.70% identified as white, 16.50% identified as African-American, 5.10% identified as Asian, 2.50% as Hispanic or Latino, and 0.70% identified as American indigenous or Alaska Native.

### Procedure

This study was approved by the Institutional Review Board (IRB) of the University of North Carolina at Chapel Hill. The participants were recruited through informational email postings at the university, community poster flyers, and Craigslist. During recruitment, 640 participants were assessed for eligibility for the study, but 325 were excluded at screening for various reasons, such as because they had prior meditation experience or because they had participated in a previous meditation study. Of the eligible participants, 84 participants could not be scheduled for their baseline assessments due to scheduling conflicts, because they did not return calls or emails, or because they were too busy. This resulted in a sample of 231 participants who were randomized to either the LKM intervention (118) or the MM (113) intervention in the larger study from which these data were derived. Of those 231 participants randomized, 217 had usable data, because a portion of participants in each condition did not complete key assessments for various reasons (e.g., never attended meditation classes or withdrew before daily reports).

After recruitment, participants first went to the laboratory for an intake session to complete a series of assessments, among which included self-compassion and depression. After that, participants were emailed surveys each day across the course of 11 weeks. This daily survey included assessments of their levels of social connectedness, as well as their positive (pride) and negative emotions (guilt and shame). In the first 2 weeks (before the beginning of the intervention), participants’ baseline levels of connectedness, positive emotions, and negative emotions were established. After that, during the following 6 weeks, participants attended either MM or LKM classes once per week. Participants were also encouraged to practice meditation at home (details below). After the meditation classes ended, participants continued completing the daily surveys, which included reporting on their emotions and social connectedness for another 3 weeks. The 11 weeks of daily reports were used to assess the change in social connectedness, pride, and guilt and shame across the interventions for people higher and lower in self-compassion, as well as the variability of these emotions within individuals on the days they practiced meditation. Depression was assessed in the first lab visit before the daily reporting of emotions started, and then again 2 weeks after the interventions ended, at the 12-week laboratory session. Although it was not part of our pre-registered analysis plan, participants also completed an assessment of depression at an 18-month follow-up assessment, and we will return to this assessment in our data analysis plan. Self-compassion was assessed in the first laboratory visit and then again at the 12-week laboratory session. McDonald’s omega reliability coefficients were calculated for all measures with 3 or more items.

The 1-h meditation classes for each participant occurred once per week, in person, with each class having a maximum of 16 people. Participants also received at-home, guided meditations, which they were encouraged to do 3–5 times per week, 20 min per day. In the case of a participant being unable to attend class in a specific week, they were told to attend the meditation class in the following week and continue their at-home meditation practice.

In the MM intervention, participants were guided throughout the 6-week intervention with the overall intention to be in the present moment, combined with a nonjudgmental acceptance of their present-moment experiences. Over the course of the 6 weeks, they progressively engaged in a series of practices which directed their conscious attention to various targets in the present moment. The targets included their breath and breathing in week 1, the body in week 2, their emotions in week 3, their thoughts in week 4, choiceless awareness in week 5, and week 6 was reserved for a final summary session in which participants reviewed and integrated the material they learned. Consistent with prior research, the MM followed the model of mindfulness proposed by Shapiro et al. (2006), where the main goal was for the individual to metacognitively observe their experiences in a non-reactive and accepting manner.

While also maintaining an attitude of acceptance and non-judgment, the goal for participants in the LKM condition was to self-generate kindhearted, compassionate feelings (Salzberg, 1995). Participants repeated a series of kindhearted phrases while directing their attention to various targets, as well as the physical sensations in their hearts. Progressively over the course of the 6 weeks, participants directed their self-generated, warmhearted feelings towards (1) a loved one, (2) themselves, (3) an acquaintance, (4) a difficult person, and (5) all beings, with week 6 being reserved for a review and integration of previous material. The main goal of the LKM was to help individuals foster their ability to feel kindness and compassion.

### Measures

#### *Daily Minutes of Meditation*

Participants provided daily reports of their meditation practice, which included (a) whether they meditated each day and (b) how many minutes they had engaged in meditation practice (on days that they did meditate). These two questions were used to construct a “minutes meditated” variable. Participants who reported they had not engaged in meditation on a particular day got a 0, and participants who reported meditating on a particular day received the number of minutes they indicated they had meditated for.

#### *Pride, Guilt, and Shame*

Pride, guilt, and shame were assessed using items from the daily version of the modified Differential Emotions Scale (Fredrickson et al., 2008). Each day, participants were asked the extent to which they felt each of these emotions over the past 24 h on a scale from 0 = *not at all* to 4 = *extremely.* As is standard for the mDES, participants were presented with three synonyms for the emotion of interest (pride: “Proud, Confident, Self-assured”; shame: “Ashamed, Humiliated, Disgraced”; guilt: “Guilty, repentant, blameworthy”). Shame and guilt were strongly positively correlated (*r* = 0.50, *p* < 0.001), and were averaged to create an overall index of participants’ shame and guilt in everyday life.

#### *Social Connectedness*

Participants’ feelings of connection to others were measured daily with the following question: “In the past 24 h, how much did you feel socially integrated or ‘on the same page’ with others?” Participants responded on a scale from 1 = *not at all* to 7 = *completely.*

#### *Depression*

Depression was measured using the Centre of Epidemiological Studies Depression (CES-D) scale (Radloff, 1977) in the first and second laboratory sessions (pre- and post-intervention). The scale had 20 items, in which participants were asked about various depressive symptoms over the last week, such as “I felt depressed” and “I felt like everything was an effort.” The items were answered on a scale from 1 = *hardly* to 4 = *most of the time*. The scale had excellent internal consistency (pre-intervention McDonald’s Omega *ω* = 0.93; post-intervention *ω* = 0.92; 18-month *ω* = 0.93).

#### *Self-compassion*

Self-compassion was measured with the short form of the self-compassion scale (Raes et al., 2011). Participants responded to 12 items (e.g., “When I fail at something important to me I become consumed by feelings of inadequacy,” or “I try to see my failings as part of the human condition”) on a scale of 1 = *almost never* to 5 = *almost always*. The internal consistency of this scale was good at pre- (*ω* = 0.87) and post-intervention (*ω* = 0.85).

### Data Analyses

All data analyses were conducted in SPSS version 29, and all data and code are provided on the Open Science Framework page for this study: https://osf.io/nu9h4/?view_only=6a9735db5fe9456b80049517bbab0047. To analyze the data in this study, we utilized multilevel modeling to account for the nested and longitudinal nature of the data. To examine whether change in daily guilt and shame, pride, and connectedness from week 1 to week 11 of the intervention was moderated by self-compassion, we implemented multilevel growth curve modeling based on the recommendations of Bolger and Laurenceau (2013). We included linear and quadratic components for time (to examine the possibility of curvilinear patterns of change in the outcomes of interest), and included a main effect for condition to account for possible differences in the outcomes of those assigned to the MM and the LKM intervention. The models included random intercepts and random slopes for the linear influence of time. Consistent with recommendations for growth curve analyses (Bolger & Laurenceau, 2013; Heck et al., 2022), we did not include random slopes for the quadratic components, because this was not an important aspect of our theoretical predictions, and because of the additional computational burden that they introduce into the models. Baseline self-compassion was included as a moderator in the linear and the quadratic components of time, so we could investigate if the trajectories of the outcomes across the intervention were different for those with higher and lower levels of baseline self-compassion. The time variable was centered around the first day of daily data collection (2 weeks prior to the intervention), MM was coded as −1, and LKM was coded as 1, and baseline self-compassion was grand mean centered. Because baseline self-compassion was mean centered, the slope for the initial analyses represents change in daily guilt and shame, pride, or connectedness at *average* levels of baseline self-compassion.

In addition to the growth curve analyses, we conducted a daily, within-person analysis to examine how daily minutes of meditation practice on a particular day predicted participants’ outcomes on that same day. For this set of analyses, 213 participants completed the measures required to be included. Consistent with recommendations for daily analyses (Bolger & Laurenceau, 2013), the variability in participants’ minutes of meditation each day was separated into two components: between-persons and within-persons. The between-person component represented each participant’s average of minutes of meditation per day across the intervention as compared to other people (i.e., the grand mean). The within-person component represented the individual’s deviations on a particular day from their own general tendency to engage in daily meditation across the intervention (i.e., within-person deviations from their person mean). We therefore examined whether baseline self-compassion interacted with both the between- and within-person components for minutes meditated in predicting the outcome variables of interest. The model was specified to include random intercepts and a random slope for the within-person minutes meditated variable (Bolger & Laurenceau, 2013). Condition was again coded as MM = −1 and LKM = 1.

To examine participants’ changes in depression, and whether these changes were different for those higher and lower in self-compassion, we conducted multilevel models according to the recommendations of West et al. (2015) to account for the longitudinal, nested nature of the data. There were 216 participants in this analysis. Each participant had two data points, one from the first laboratory visit at week 1, and another from the second laboratory visit at week 12. The models were specified to include (a) a main effect of baseline self-compassion on depression to investigate if self-compassion predicted lower overall depression, (b) a main effect of time (before and after intervention, coded as 0 and 1) on depression, to examine whether participants’ levels of depression changed from the first visit to the laboratory to the second visit, (c) a main effect of condition (coded as −1 for MM and 1 for LKM), (d) the interaction between time (before and after the intervention) and self-compassion, (e) the interaction between condition and self-compassion, (f) the interaction between the time (before and after the intervention) and condition, and (g) a three-way interaction between time, condition (MM and LKM) and baseline self-compassion. The key parameter of interest was the interaction between time and self-compassion, or whether participants differentially experienced change from pre- to post-intervention depending on their levels of baseline self-compassion. All continuous variables were grand mean-centered before we conducted analyses. Although we attempted to specify a random slope for the time variable, when we specified this random slope, the model failed to converge. As such, in the final model, we included only random intercepts. Because baseline self-compassion was mean centered, the coefficient for time in the initial analyses represented change in daily depression at *average* levels of baseline self-compassion.

To test whether the indirect effect of time → changes in self-compassion → changes in depression was moderated by baseline levels of self-compassion, we conducted multilevel moderated mediation analyses using the MLmed macro in SPSS (Hayes & Rockwood, 2020). MLmed uses Monte Carlo simulation to compute unbiased confidence intervals for indirect effects within multilevel models. The MLmed macro provides an estimate of moderated mediation, and the hypothesized pattern of findings and model specification is presented in Online Resource 5 on the OSM.

Although our primary, pre-registered analyses examined changes from the pre-intervention lab visit to the post-intervention lab visit, our study also contained depression data from an 18-month follow-up laboratory visit. In a supplemental, non-preregistered analysis, we conducted a multilevel analysis in which we examined change from pre-intervention to the lab visits at (a) post-intervention and (b) 18-month follow-up, and whether this depended on baseline levels of self-compassion. As in the above analysis, we tested whether any change in trait depression differed depending on condition (MM vs. LK). We also attempted to include random slopes in this model for the within-person time variables, but when we included these random slopes, the model failed to converge, so we specified the model with random intercepts only.

We tested for residual normality and residual independence by extracting residuals from our primary analyses, and then visually inspecting them using histograms and qq-plots, as well as examining correlations between predictors and residuals. Residual normality appeared satisfactory for all primary analyses, and we note that multilevel analyses are often robust to violations of distributional assumptions (Schielzeth et al., 2020).

#### Missing Data

For two of the three sets of pre-registered analyses—the growth curve and pre-post analyses—participants were only missing data on the outcome variables. In these analyses, for the final sample of interest in each analysis, all participants had complete data on all predictor variables, including baseline self-compassion, the time indicator variables, condition, and the interactions between these variables. As noted by Enders (2022), multilevel modeling with restricted maximum likelihood (REML) tends to encounter issues with biased estimates when data is missing on *both* predictors and outcomes. However, when data is only missing on outcomes, multilevel modeling with REML is capable of flexibly incorporating participants with missing data into analyses (Enders, 2022). As such, in the growth curve and pre-post analyses, we used restricted maximum likelihood (REML) estimation in our models and incorporated all participants who had missing data on outcome variables into our analyses.

In the within-person analyses, one of our primary predictor variables (daily minutes of meditation) also included missing data. In this case, we estimated the primary models using REML in SPSS, but replicated all models using FIML estimation in MPlus and examined whether there were any substantive differences between the two sets of analyses. FIML allows for flexible incorporation of missing data on both predictors and outcome variables, so it is useful as a sensitivity analysis in situations where data is missing on both predictors and outcomes.

### Statistical Power

Power analyses were conducted a priori for the original study on which these data are derived. We also conducted power analyses for this particular paper using the method and power calculator provided by Murayama et al. (2022). Murayama et al. (2022) suggest that power for two-level multilevel models can be adequately calculated using summary statistics, as compared to more laborious and intensive methods, such as Monte Carlo simulations. Using the effect size estimates from the daily guilt and shame analyses and the calculator provided by Murayama et al. (2022), results of a power analysis suggest that a sample of only 176 participants would be needed to achieve a power of 0.80, assuming small to moderate-sized effects. For the guilt and shame analyses, we had a sample of *n* = 217 people, and *k* = 12,262 daily observations. Thus, our analyses appeared to be adequately powered.

## Results

Descriptive statistics and correlations are presented in Online Resource 1 in the OSM. Consistent with prior research, at the descriptive level, participants’ levels of guilt and shame appeared lower during and after the intervention. Also consistent with past research, participants decreased depression from before to after the intervention. In addition, participants’ levels of social connectedness increased from before the intervention to during and after the intervention. However, no clear pattern emerged with respect to participants’ pride.

### Self-compassion and Daily Negative Emotions Across the Intervention

Results of multilevel growth curve analyses examining whether baseline self-compassion moderated longitudinal patterns of change in daily guilt and shame across the intervention are presented in Table 1. The linear coefficient for time was negative and significant (*B* = −0.006, *p* < 0.001), and the quadratic coefficient was significant and positive (*B* = 0.00006, *p* < 0.001), which suggests a curvilinear pattern of change whereby at average levels of baseline self-compassion participants tended to decrease in guilt and shame at the beginning of the intervention, a pattern of change which then tapered off after the end of the intervention. The main effect of self-compassion on guilt and shame was also negative and significant, meaning participants with higher levels of self-compassion generally reported lower levels of guilt and shame across the study (*B* =—0.24, *p* < 0.001). The main effect of condition (MM or LKM) was not significant, meaning participants in the two meditation conditions were not significantly different in daily guilt and shame. Finally, and central to our hypotheses, the interactions between the linear and quadratic components for time and baseline self-compassion were both significant in predicting levels of guilt and shame.

**Table 1:** Results of multilevel growth curve analyses investigating levels of negative and positive emotions during the intervention period for people higher and lower in self-compassion

Predictor	Guilt and shame					Pride				*r*
	*B*	*p*	95% CI		*r*	*B*	*p*	95% CI		
			Lower	Upper				Lower	Upper	
Intercept	0.48	< 0.001	0.42	0.54	-	1.93	< 0.001	1.87	2.04	-
Time	−0.006	< 0.001	−0.008	−0.004	0.12	−0.01	< 0.001	−0.012	−0.008	0.17
Time^2^	0.00006	< 0.001	0.00004	0.00008	0.10	0.0001	< 0.001	0.0001	0.00008	0.16
SC	−0.24	< 0.001	−0.33	−0.16	0.33	0.43	< 0.001	0.28	0.58	0.33
Cond	−0.01	0.854	−0.06	0.05	0.01	−0.09	0.11	−0.19	0.02	0.11
Day × SC	0.004	0.002	0.002	0.006	0.05	−0.002	0.21	−0.006	0.001	0.02
Time^2^ × SC	−0.00003	0.013	−0.00007	−0.000008	0.04	−0.000002	0.91	−0.00004	0.00004	0.00

*SC*, self-compassion; *CI*, confidence interval. *r* was calculated using the method used by Kashdan and Steger (2006): *r* = √(*t*^2^/*t*^2^ + *df*). For the guilt and shame analysis, *n* = 217, *k* = 12,262. For the pride analysis, *n* = 217, *k* = 12,580

**Fig. 1:**
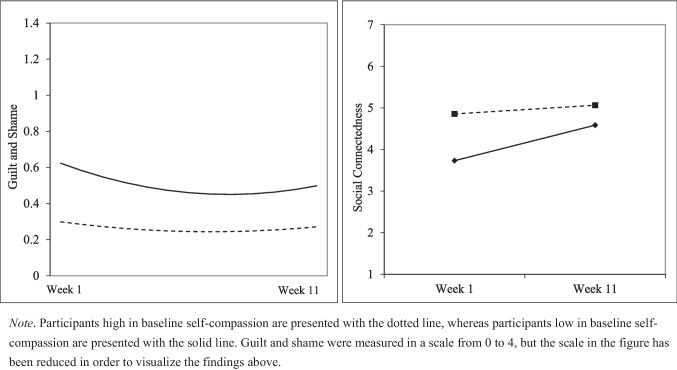
Multilevel growth analyses across the intervention from week 1 to week 12 for guilt and shame and social connectedness

To decompose the significant interactions, simple slope analyses were conducted to investigate the changes in participants’ guilt and shame at high (+ 1 *SD*) and low (−1 *SD*) levels of baseline self-compassion, and the interaction is presented in Fig. 1. With respect to the linear pattern of change, although participants with higher (*B* = −0.003, *p* = 0.008, 95% CI [−0.006, −0.001]) and lower levels (*B* = −0.009, *p* < 0.001, 95% CI [−0.012, −0.007]) of baseline self-compassion experienced a significant decrease in levels of guilt and shame across the early portion of the intervention, those with lower levels of baseline self-compassion experienced a stronger decrease. With respect to the quadratic interaction, those higher in baseline self-compassion showed less of a tendency to increase in guilt in shame at the end of the intervention (*B* = 0.00003, *p* = 0.02, 95% CI [0.00004, 0.000007]) as compared to those lower in baseline self-compassion (*B* = 0.00009,* p* < 0.001, 95% CI [0.00006 and 0.0001]).

### Self-compassion and Daily Pride Across the Intervention

Results of multilevel growth curve analyses examining whether baseline self-compassion moderated longitudinal patterns of change in daily pride across the intervention are presented on the right side of Table 1. Surprisingly, linear and quadratic components for time were both significant, whereby at average levels of baseline self-compassion participants’ levels of pride decreased across the intervention but then started to go back up once the intervention ended. Finally, the main effect of self-compassion was statistically significant (*B* = 0.43, *p* < 0.001), such that participants with greater baseline self-compassion tended to have greater pride across the intervention. The main effect of condition was not significant. Additionally, contrary to our hypotheses, the interactions between self-compassion and the linear and quadratic components for time were both not significant, suggesting that different levels of baseline self-compassion were not associated with how participants responded to the meditations in terms of pride.

### Self-compassion and Affective Experiences in Response to Meditation on a Particular Day

Next, we conducted a series of within-person analyses to examine whether baseline self-compassion moderated the link between daily engagement in meditation practice and participants’ affective outcomes. Results for both (a) guilt and shame and (b) pride are in Online Resource 2 in the OSM. The key interactions between baseline self-compassion and minutes meditating at the between- and within-person levels were not significant in predicting guilt and shame. Similarly, the interactions between self-compassion and daily minutes meditated at the between- and within-person levels were not significant. Thus, contrary to our hypotheses, baseline self-compassion did not moderate the association between daily engagement in meditation and daily guilt and shame or pride. We re-conducted these analyses using FIML estimation in MPlus and the substantive conclusions were identical to the results presented in Online Resource 2.

### Social Connectedness Across the Intervention

The multilevel growth curve model examining social connectedness across the course of the intervention period (Table 2) revealed a positive and significant main effect of the linear slope for time, whereby participants with average levels of baseline self-compassion showed an increase in social connectedness across the intervention (*B* = 0.007, *p* < 0.001). The quadratic component of time was not statistically significant (*p* = 0.243), indicating a linear pattern of change in connectedness. Consistent with predictions, the interaction between the linear component for time and baseline self-compassion was statistically significant (*B* = −0.006, *p* = 0.026). This interaction is plotted in the right panel of Fig. 1.

**Table 2:** Results of multilevel growth curve analysis investigating levels of connectedness across the interventions period for people higher versus lower in self-compassion

Predictor	*B*	*p*	95% CI		*r*
			Lower	Upper	
Intercept	4.32	< 0.001	4.16	4.47	-
Time	0.007	< 0.001	0.003	0.01	0.09
Time^2^	−0.00002	0.24	−0.00006	0.00001	0.02
Self-compassion	0.80	< 0.001	0.58	1.01	0.42
Condition (MM or LKM)	0.03	0.71	−0.12	0.17	0.03
Time × SC	−0.006	0.026	−0.01	−0.001	0.05
Time^2^ × SC	0.00005	0.074	−0.000005	0.0001	0.03

*SC*, self-compassion; *CI*, confidence intervals. *r* was calculated using the method used by Kashdan and Steger (2006): *r* = √(*t*^2^/*t*^2^ + *df*). *n* = 217, *k* = 12,581

To decompose this interaction, we conducted simple slopes analyses at low and high levels (± 1 *SD*) of baseline self-compassion. Results demonstrated that participants with lower levels of self-compassion significantly increased in social connectedness across the intervention (*B* = 0.011, *p* < 0.001, 95% CI [0.006, 0.016]), whereas those with higher levels of baseline self-compassion did not experience significant changes in levels of connectedness (*B* = 0.003, *p* = 0.25, 95% CI [−0.002, 0.01]). Thus, participants with both low and average levels of baseline self-compassion experienced increases in social connectedness across time, but those with high levels of baseline self-compassion did not.

### Daily Social Connectedness

We again conducted a within-person analysis to examine whether baseline self-compassion moderated response to daily meditation practice on a particular day. Results are presented in Online Resource 3 in the OSM. The greater minutes meditated variable was significantly associated with greater daily social connectedness at the within-person level, but not at the between-person level. However, the interactions between baseline self-compassion and minutes meditated at both the within-person and between-person levels were not statistically significant, meaning baseline self-compassion did not alter the between-person or within-person link between minutes of meditation practice and feelings of social connectedness on a particular day. We again re-conducted this analysis using FIML estimation in MPlus and the substantive conclusions were identical to those presented in Online Resource 3.

### Changes in Depression Pre- to Post-intervention

Next, we conducted a multilevel analysis to examine whether baseline levels of self-compassion moderated changes in depression from before to after the meditation training, and the results of this analysis are presented in Table 3. At average levels of baseline self-compassion, participants demonstrated a marginally significant decrease from pre- to post-intervention in depression (*B* = −0.02, *p* = 0.10). The predicted interaction between baseline self-compassion and time was statistically significant in predicting depression (*B* = 0.05, *p* = 0.02).

**Table 3:** Results of multilevel analysis examining self-compassion as a moderator of change in depression from pre- to post-meditation training

Predictor	*B*	*SE*	*p*	LLCI	ULCI	*r*
Intercept	1.59	0.02	< 0.001	1.54	1.64	-
Time	−0.02	0.01	0.10	−0.05	0.004	0.12
SC	−0.28	0.03	< 0.001	−0.35	−0.22	0.49
Condition (MM or LKM)	0.02	0.02	0.40	−0.03	0.07	0.06
Time × SC	0.05	0.02	0.02	0.01	0.09	0.16
Condition × SC	−0.04	0.03	0.24	−0.11	0.03	0.08
Time × condition	0.01	0.01	0.43	−0.02	0.04	0.06
Time × condition × SC	−0.02	0.02	0.43	−0.06	0.02	0.06

*SC*, self-compassion. Time is an indicator variable that refers to before or after the meditation intervention. *LLCI*, lower level of the 95% confidence interval; *ULCI*, upper level of the 95% confidence interval. *r* was calculated using the method used by Kashdan and Steger (2006): *r* = √(*t*^2^/*t*^2^ + *df*). *n* = 216 people

**Fig. 2:**
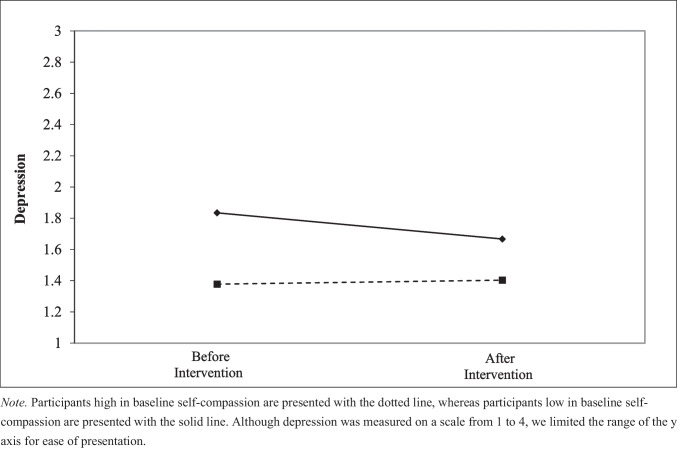
Pre-post analysis of levels of depression before and after the intervention for those with lower and higher levels of self-compassion

To decompose this interaction—which is plotted in Fig. 2—we conducted simple slopes analysis at low (−1 *SD*) and high (+ 1 *SD*) levels of baseline self-compassion. Consistent with our predictions, we found that the reduction in depression from pre- to post-intervention was significant for those with low levels of self-compassion (*B* = −0.06, *p* = 0.006), but not significant for those with high baseline levels of self-compassion (*B* = 0.01, *p* = 0.56).

### Moderated Mediation Analyses

We conducted moderated mediation analysis using the MLMed Macro (Hayes & Rockwood, 2020) to examine whether baseline levels of self-compassion moderated the indirect effect of time (pre- to post-intervention) → enhanced self-compassion → reduced depression. Results of this analysis are presented in Table 4 and displayed in the Online Resource 6 in the OSM.

**Table 4:** Results of moderated mediation analysis

Outcome	Predictor	*B*	*p*	95%CI		*r*
				Lower	Upper	
SC	Intercept	3.23	< 0.001	3.18	3.27	-
	Time	0.19	< 0.001	0.13	0.26	0.37
	Baseline SC	0.81	< 0.001	0.76	0.85	0.92
	Condition (MM or LKM)	−0.01	0.75	−0.07	0.05	0.02
	Baseline SC × time	−0.39	< 0.001	−0.48	−0.30	0.50
Outcome	Predictor	*B*	*p*	Lower	Upper	*r*
Depression	Intercept	1.58	< 0.001	1.49	1.66	-
	Time	−0.002	0.93	−0.06	0.06	0.01
	Self-compassion	−0.19	< 0.001	−0.30	−0.08	0.25
	Condition (MM or LKM)	0.05	0.36	−0.06	0.17	0.06

Index of moderation mediation (*estimate* = 0.07, 95% CI [0.03, 0.12]). Time is an indicator variable that refers to before or after the meditation intervention. *SC*, self-compassion; *CI*, confidence intervals

With respect to the *a* path for the mediation model, time was a significant and positive predictor of self-compassion across the intervention (*B* = 0.19, *p* < 0.001), indicating participants’ levels of self-compassion increased after the meditation intervention. Not surprisingly, baseline self-compassion also strongly predicted participants’ levels of post-intervention self-compassion (*B* = 0.81, *p* < 0.001). Additionally, the interaction between baseline self-compassion and time was statistically significant. Because the interaction is negative, this demonstrates that the lower one’s baseline self-compassion, the more they reported increases in self-compassion after the intervention. With respect to the *b* path of the mediation, self-compassion was negatively associated with depression (*B* = −0.19, *p* < 0.001). Consistent with evidence for mediation, the association between time and depression was not significant when including self-compassion (the mediator) in the model.

Consistent with expectations, the index of moderated mediation was statistically significant (*estimate* = 0.07, 95% CI [0.03, 0.12]). To decompose the interaction, we examined the indirect effect of time → changes in self-compassion → depression at low (−1 *SD*) and high levels (+ 1 *SD*) of baseline self-compassion. When baseline levels of self-compassion were low, the indirect effect of time → increased self-compassion → lower depression was statistically significant (*estimate* = −0.09, *SE* = 0.028, 95% CI [−0.14, −0.04], *p* = 0.001). By contrast, for those high in baseline self-compassion, the indirect effect of time → greater self-compassion → lower depression was not significant (*estimate* = 0.02, *SE* = 0.01, 95% CI [0.0009, 0.042], *p* = 0.08). Thus, people low in baseline self-compassion tend to experience greater increases in self-compassion after the intervention, which was then associated with lower depression. By contrast, the indirect link between time and depression via greater self-compassion was not significant for those higher in baseline self-compassion.

### Changes in Depression at 18-Month Follow-Up

Results of a multilevel analysis examining change from pre-intervention to (a) post-intervention, and (b) the 18-month follow-up are presented in Online Resource 4 in the OSM. The interaction between baseline self-compassion and the post-intervention indicator variable was once again significant, demonstrating that change from pre- to post-intervention depended on baseline levels of self-compassion. Notably, the interaction between baseline self-compassion and the 18-month indicator variable was also significant, demonstrating that change from pre-intervention to the 18-month follow-up time point also depended on baseline self-compassion.

To decompose this interaction, we conducted simple slopes analyses at low (−1 *SD*) and high (+ 1 *SD*) levels of baseline self-compassion. Results are also presented in Online Resource 7 in the OSM. When participants were low in baseline self-compassion, they reported significant decreases from pre-intervention to post-intervention (*B* = −0.13, *p* = 0.003), and from pre-intervention to the 18-month follow-up (*B* = −0.11, *p* = 0.014) lab visits. When participants were high in baseline self-compassion, they did not experience change in depression from pre-intervention to post-intervention (*B* = 0.03, *p* = 0.50), or from pre-intervention to the 18-month follow-up (*B* = 0.04, *p* = 0.30).

## Discussion

Drawing on data from a randomized intervention of over 200 adults, we examined whether individual differences in self-compassion prior to meditation training were associated with better affective, social, and mental health outcomes in response to meditation training. Consistent with our pre-registered predictions, across the course of the intervention, people with lower baseline self-compassion (as compared to those with higher levels of self-compassion) experienced (a) greater decreases in guilt and shame, (b) greater reductions in depression, and (c) greater increases in social connectedness. Contrary to our predictions, baseline self-compassion was not associated with participants’ responses to engagement in meditation on a single day of practice. In addition, consistent with our theorizing, moderated mediation analyses demonstrated that the greater decreases in depression experienced by people with low baseline self-compassion were mediated by their greater increases in self-compassion from pre- to post-intervention.

One of the core findings of this research is that, across the course of MM and LKM intervention, those with low self-compassion experience especially prominent decreases in guilt, shame, and depression as compared to those with high self-compassion. Guilt, shame, and depression are often a product of negative self-talk (Tangney & Dearing, 2002; Thompson et al., 2020) and rumination around failures (Liu et al., 2022; Mosewich et al., 2011). As such, our findings suggest that these forms of meditation are especially helpful in addressing the challenges that people who are low in self-compassion face with negative self-talk and rumination. By contrast, people with high baseline levels of self-compassion likely already had lower levels of negative self-talk and rumination that tends to be associated with daily guilt and shame, and therefore likely had less room to benefit from MM and LKM.

Although our primary pre-registered analyses examined changes in depression from pre- to post-intervention, we also conducted supplemental analyses using depression at an 18-month follow-up laboratory visit. Results from this analysis demonstrated that, compared to participants high in baseline self-compassion, participants low in baseline self-compassion continued to show greater decreases in depression from pre-intervention to the 18-month follow-up. In other words, the beneficial change in depression that people low in baseline self-compassion experienced was sustained even months after the end of the intervention, whereas people high in baseline self-compassion continued to report no change in depression.

We similarly found that those with lower baseline self-compassion experienced greater increases in social connectedness during the MM and LKM training, as compared to those with higher baseline self-compassion. It is likely that when those with lower self-compassion practice MM and LKM they tend to experience particularly strong increases in compassion towards and acceptance of themselves, and as a result feel more equipped to connect with others. By contrast, people high in baseline levels of self-compassion are already skilled in self-acceptance, mindfulness, and empathy, skills which tend to equip them to connect with others (Gilbert & Procter, 2006; Neff, 2023). As such, people high in self-compassion appear less likely to benefit in terms of social connectedness from the skills being cultivated in meditation practice. We note here, again, that although we registered the UCLA Loneliness Scale as an outcome, we were unable to examine this scale because it was unexpectedly not available in the larger underlying study. Because of this, we used daily social connectedness as an outcome.

Additionally, our moderated mediation analyses not only extend our moderation hypothesis to a clinically relevant outcome (depression), but also identify the theorized mediator (enhanced self-compassion), which provides a potential explanatory mechanism for the findings in this work. Specifically, we theorized that people who are low in self-compassion have the most to gain from MM and LKM, which explains why they tend to report greater benefits from meditation across time. Our moderated mediation analyses suggest this explanation is plausible. We note that we were unable to conduct mediation analyses in daily life because self-compassion was not assessed at the daily level, but we suspect that if daily self-compassion was assessed, it would similarly mediate the link between change across the course of the intervention and other key outcomes like guilt, shame, and social connectedness.

Although baseline self-compassion was generally associated with greater levels of daily pride (i.e., we found a significant main effect), contrary to hypotheses, baseline self-compassion was not associated with how people responded to each meditation regarding pride at the daily level or across time. One explanation for this is our assessment of pride. Prior research documents that pride can be divided into two distinct types: authentic and hubristic pride (Tracy & Robins, 2004). Authentic pride refers to the pride that arises from accomplishments and the recognition of one’s efforts (Weidman et al., 2016; Wubben et al., 2012). In contrast, hubristic pride is the type of feeling that emerges from one’s global beliefs about their abilities, being more related to neuroticism and defensiveness (Carver et al., 2010; Tracy et al., 2009). In this study, we did not assess authentic and hubristic pride separately, which may explain why we did not observe differences in how people low versus high in self-compassion responded in terms of pride. That is, levels of self-compassion could be associated with how people respond to MM and LKM in terms of authentic but not hubristic pride. For instance, enhanced self-compassion associated with meditation training may encourage people to become more capable of recognizing their genuine accomplishments, which may be particularly helpful for people with low baseline levels of self-compassion. However, because we did not assess authentic versus hubristic pride, we were not able to test this possibility*.*

While we largely discussed each of these outcomes (depression, shame and guilt, social connectedness) separately, the way in which individuals with low self-compassion derive greater benefit from meditation likely reflects a broader regulatory process. For instance, scholars have argued that enhanced self-compassion is a crucial emotional regulatory skill for those prone to experiencing depression and shame (e.g., Gilbert & Procter, 2006; Gilbert, 2014). Thus, the improved outcomes observed among individuals with low baseline self-compassion may be part of a larger process driven by enhanced emotional regulation in response to meditation training.

We also predicted that self-compassion would be associated with people’s responses to MM and LKM on particular days of meditation practice; however, daily dose-response analyses demonstrated this was not the case. One explanation for this finding is that people with lower levels of self-compassion require time to learn the skills that are associated with decreases in guilt, shame, and depression, as well as greater connectedness. That is, we think self-compassion is a skill that is learned over time—one that is unlikely to improve significantly after only one session of either MM or LKM. We note, of course, that without a control group, our data are only correlational, but we speculate here on the potential reasons for our findings in order to understand the discrepancies between the daily dose-response analyses and the longitudinal, growth curve analyses.

Although we tested for differences between MM and LKM throughout our analyses, baseline self-compassion acted as a moderator of response to the meditation intervention regardless of whether participants engaged in MM or LKM. We suspect this finding is due to the fact that, although they are substantively different practices, both MM and LKM tend to cultivate compassion (e.g., Boellinghaus et al., 2014). Whereas in LKM people actively generate kindhearted feelings toward themselves and other beings, in MM people learn to view their present-moment experiences in a kind, accepting way. Our results suggest that both of these practices tend to be particularly beneficial for individuals low in self-compassion, who struggle with self-kindness.

Although they were not the primary focus of our analyses, we note additionally that individuals with *average* levels of baseline self-compassion tended to experience beneficial changes on some of the key outcomes that we examined. For instance, as compared to individuals with high baseline self-compassion, individuals with average levels of baseline self-compassion experienced beneficial changes in guilt, shame, and social connectedness, as well as marginally significant decreases in depression from pre- to post-intervention. This is consistent with the large body of literature demonstrating that meditation interventions tend to have broadly beneficial effects (e.g., Goldberg et al., 2022). Thus, while our results document individual differences influencing how people respond to meditation interventions, and that individuals *low* in self-compassion are especially likely to experience benefits in response to meditation training, they equally demonstrate that people with average levels of self-compassion tend to benefit as well.

These findings have practical implications. Although meditation interventions have an extensive body of support for a broad range of outcomes, the effect size of these interventions is not always the same for all people or outcomes (e.g., Goldberg et al., 2022). Given that our work demonstrates that baseline levels of self-compassion influence the extent to which people experience beneficial outcomes in response to meditation, clinicians may be able to use individual difference characteristics like self-compassion as a potential screening tool to determine who is most likely to benefit from these meditation practices. Moreover, if the evidence is communicated properly, individuals who actively seek a psychological wellness tool from a range of available options (e.g., daily gratitude writing tasks, meditation, etc.) may be able to choose one in an informed manner based on knowledge of their own traits (i.e., people who know they are low in self-compassion could be directed towards MM and LKM via science communication). We note that practical applications should move forward with caution because these findings require replication. We also note that other research has demonstrated that other individual difference factors influence how people respond to meditation interventions (Buric et al., 2022, 2025), and so self-compassion should be considered as part of a range of factors that may contribute to the way people respond to meditation. This study also has many strengths. For instance, participants reported their daily emotions every day across the course of 11 weeks, which a) allowed us to analyze the longitudinal course of their emotions and (b) tends to be less prone to retrospective bias (Bolger et al., 2003; Schwarz 2007). We also drew on a large sample of participants drawn from the community who completed a validated, randomized meditation training.

### Limitations and Future Directions

Although this research has many strengths, it is also important to mention its limitations. One of them is that this study did not have a control condition of participants who were not trained in meditation, which limits our ability to draw causal conclusions. It could be the case that participants in the MM and LKM groups experienced changes in levels of depression, guilt and shame, and connectedness due to placebo or expectancy effects in response to meditation training. Notably, however, given that extensive prior research has been devoted to establishing the efficacy of meditation training beyond active controls (Goldberg et al., 2022; Goyal et al., 2014), the goal of this work was not to establish whether MM and LKM are effective. Instead, this research focused on examining individual differences in how people respond to MM and LKM. Still, confounds could interact with the moderator and introduce bias in the estimation of interventions (Marsden et al., 2022). In addition, participants in this study were from a specific part of the USA, predominantly White, and they were all midlife adults interested in meditation practices. Consequently, the results may be different in other regions of the USA, in other parts of the world, or with a different demographic sample, and future research should investigate this possibility. Additionally, we note that many well-established meditation interventions, such as Mindfulness-Based Stress Reduction (e.g., Hoge et al., 2023), tend to last 8 weeks, whereas our intervention only lasted 6 weeks. This presents challenges with directly comparing, reproducing, and implementing our intervention, as compared with these other interventions.

This study investigated how different levels of self-compassion influenced the outcomes of participants who trained in either mindfulness meditation or loving-kindness meditation, providing evidence that people low in self-compassion experienced greater benefits than those higher in baseline self-compassion. More specifically, we found that those with lower self-compassion showed more pronounced decreases in guilt, shame, and depression, and more pronounced increases in social connectedness across the intervention, but not after a particular day engaging in meditation. We also uncovered that the decreases in depression before and after the intervention were mediated by an increase in participants’ self-compassion levels. These findings highlight the relevance of individual differences in how people respond to MM and LKM training and also contribute to understanding how meditation works. Additionally, this work demonstrated that these individual differences in how people respond to MM and LKM persisted even at an 18-month follow-up. Building on this work, future research should continue to explore the role of individual differences in response to MM and LKM, so we can better understand *for whom* these practices are most beneficial.

## Supplementary Information

Below is the link to the electronic supplementary material.

ESM1 (DOCX 131 KB)

## Data Availability

All data analyses were conducted in SPSS version 29 and MPlus version 8, and all data and code are provided on the Open Science Framework page for this study, https://osf.io/nu9h4/?view_only=6a9735db5fe9456b80049517bbab0047.
