# Supplementary material for: Understanding the Affective and Mental Health Outcomes of Meditation Interventions: The Role of Individual Differences in Self-compassion
Source: Mindfulness (N Y). 2026 Apr 8;17(5):1497–513. doi: 10.1007/s12671-026-02803-z (PMC13180764; doi:10.1007/s12671-026-02803-z)
Supplement: Supplementary file 1 — (DOCX 131 KB) [file 12671_2026_2803_MOESM1_ESM.docx]

**Online Resource 1**

*Descriptive Statistics of Each Variable Before, During and After MM and LKM Interventions*

|  | Before Intervention | | | | During Intervention | | | | After Intervention | | | |
| --- | --- | --- | --- | --- | --- | --- | --- | --- | --- | --- | --- | --- |
|  | MM | | LKM | | MM | | LKM | | MM | | LKM | |
| Outcomes | *M* | *SD* | *M* | *SD* | *M* | *SD* | *M* | *SD* | *M* | *SD* | *M* | *SD* |
| Guilt and shame | 0.41 | 0.73 | 0.39 | 0.65 | 0.33 | 0.62 | 0.35 | 0.62 | 0.31 | 0.58 | 0.32 | 0.60 |
| Pride | 1.93 | 1.1 | 1.77 | 1.1 | 1.84 | 1.1 | 1.66 | 1.04 | 1.93 | 1.09 | 1.79 | 1.06 |
| Connectedness | 4.34 | 1.56 | 4.37 | 1.48 | 4.51 | 1.46 | 4.56 | 1.43 | 4.71 | 1.42 | 4.68 | 1.45 |
| Depression | 1.60 | 0.49 | 1.63 | 0.47 | . | . | . | . | 1.52 | 0.43 | 1.60 | 0.43 |
| Self-compassion | 3.13 | 0.78 | 3.12 | 0.70 | . | . | . | . | 3.34 | 0.71 | 3.32 | 0.57 |

*Note.* MM, mindfulness meditation. LKM, loving-kindness meditation. *M*, mean. *SD*, standard deviation. Before intervention = two weeks before meditation training for guilt and shame, pride and connectedness, and three weeks before the intervention in the first laboratory visit for depression and self-compassion. During the intervention = six weeks during MM or LKM training. After intervention = three weeks following the intervention for guilt and shame, pride and connectedness and four weeks after intervention at second lab visit for depression and self-compassion.

**Online Resource 2**

*Results of Multilevel Analyses Examining Guilt, Shame, and Pride in Responses to Meditation on a Particular Day*

|  | Guilt and Shame | | | | | Pride | | | | |
| --- | --- | --- | --- | --- | --- | --- | --- | --- | --- | --- |
| Predictor | *B* | *p* | 95% CI | |  | *B* | *p* | 95% CI | |  |
|  |  |  | Lower | Upper |  |  |  | Lower | Upper |  |
| Intercept | 0.47 | <0.001 | 0.41 | 0.54 |  | 1.78 | <0.001 | 1.65 | 1.90 |  |
| Self-compassion | -0.16 | <0.001 | -0.23 | -0.08 |  | 0.33 | <0.001 | 0.17 | 0.49 |  |
| Cond (MM or LKM) | 0.000 | 0.99 | -0.06 | 0.06 |  | -0.11 | 0.06 | -0.22 | 0.00 |  |
| MinsMedBP | -0.001 | 0.83 | -0.01 | 0.01 |  | 0.01 | 0.25 | -0.01 | 0.02 |  |
| MinsMedWP | -0.001 | 0.01 | -0.002 | 0.000 |  | 0.00 | 0.81 | -0.002 | 0.001 |  |
| SC*MinsMedBP | 0.001 | 0.84 | -0.01 | 0.01 |  | -0.004 | 0.74 | -0.03 | 0.02 |  |
| SC*MinsMedWP | 0.000 | 0.51 | -0.002 | 0.001 |  | -0.001 | 0.62 | -0.003 | 0.002 |  |
| Day | -0.003 | <0.001 | -0.004 | -0.002 |  | 0.00 | 0.62 | -0.002 | 0.001 |  |

*Note.*SC, self-compassion. MinsMedBP = minutes meditated between person. MinsMedWP = minutes meditated within person. CI = confidence intervals.

**Online Resource 3**

*Results of Within-Person Analysis Investigating Levels of Daily Social Connectedness*

| Parameter | *B* | *p* | 95% CI | |
| --- | --- | --- | --- | --- |
|  |  |  | Lower | Upper |
| Intercept | 4.31 | <0.001 | 4.14 | 4.48 |
| SC | 0.66 | <0.001 | 0.44 | 0.88 |
| Condition (MM and LKM) | 0.03 | 0.74 | -0.13 | 0.18 |
| MinsMedBP | 0.01 | 0.26 | -0.01 | 0.03 |
| MinsMedWP | 0.002 | 0.01 | 0.001 | 0.004 |
| SC*MinsMedBP | 0.002 | 0.88 | -0.03 | 0.03 |
| SC *MinsMedWP | -0.002 | 0.17 | -0.01 | 0.00 |
| Day | 0.01 | <0.001 | 0.01 | 0.01 |

*Note.* SC, self-compassion. MinsMedBP, minutes meditated between person. MinsMedWP, minutes meditated within person. CI, confidence intervals.

**Online Resource 4**

*Multilevel analysis examining change in depression from pre-intervention to the post-intervention and 18-month follow-up time points*

| Predictor | *B* | *SE* | p | *LLCI* | *ULCI* | *r* |
| --- | --- | --- | --- | --- | --- | --- |
| Intercept | 1.61 | 0.04 | <0.001 | 1.52 | 1.69 | - |
| Post-Intervention | -0.07 | 0.04 | 0.08 | -0.15 | 0.01 | 0.09 |
| 18-Months | -0.04 | 0.04 | 0.30 | -0.13 | 0.04 | 0.05 |
| Self-Compassion GMC | -0.30 | 0.05 | <0.001 | -0.40 | -0.20 | 0.32 |
| Condition | 0.02 | 0.06 | 0.77 | -0.10 | 0.13 | 0.02 |
| Post-Intervention * SC | 0.10 | 0.04 | 0.01 | 0.02 | 0.18 | 0.13 |
| 18-Months * SC | 0.10 | 0.04 | 0.01 | 0.02 | 0.19 | 0.12 |
| Condition x SC | -0.07 | 0.07 | 0.27 | -0.21 | 0.06 | 0.07 |
| Post-Intervention * Condition | 0.04 | 0.06 | 0.46 | -0.07 | 0.16 | 0.04 |
| 18-Months * Condition | 0.02 | 0.06 | 0.72 | -0.10 | 0.14 | 0.02 |

*Note*. *n* = 216. When baseline levels of self-compassion were low (-1 *SD*), participants experienced decreases in depression from pre-intervention to post-intervention (*B* = -0.13, *p* = .003, *r* = .15), and from pre-intervention to the 18-months intervention (*B* = -0.11, *p* = 0.014, *r* = .12). When baseline levels of self-compassion were high, participants did not experience change from pre-intervention to post-intervention (*B* = 0.03, *p* = .50, *r* = 0.04), or from pre-intervention to the 18-month follow-up (*B* = 0.04, *p* = 0.30, *r* = .05). The above analysis was specified with random intercepts only. Although we attempted to include random slopes for the within-person time variables (post-intervention and 18-month follow-up), the model failed to converge when we did so. SC = self-compassion. GMC = grand mean centered. LLCI = lower level of the 95% confidence interval. ULCI = upper level of the 95% confidence interval.

**Online Resource 5**

*Hypothesized Moderated Mediation Model*

Change in Depression

Time (Pre vs post intervention)

Change in Self-Compassion

Baseline Self-Compassion

**Online Resource 6**

*Overview of Results of Moderated Mediation Analyses*


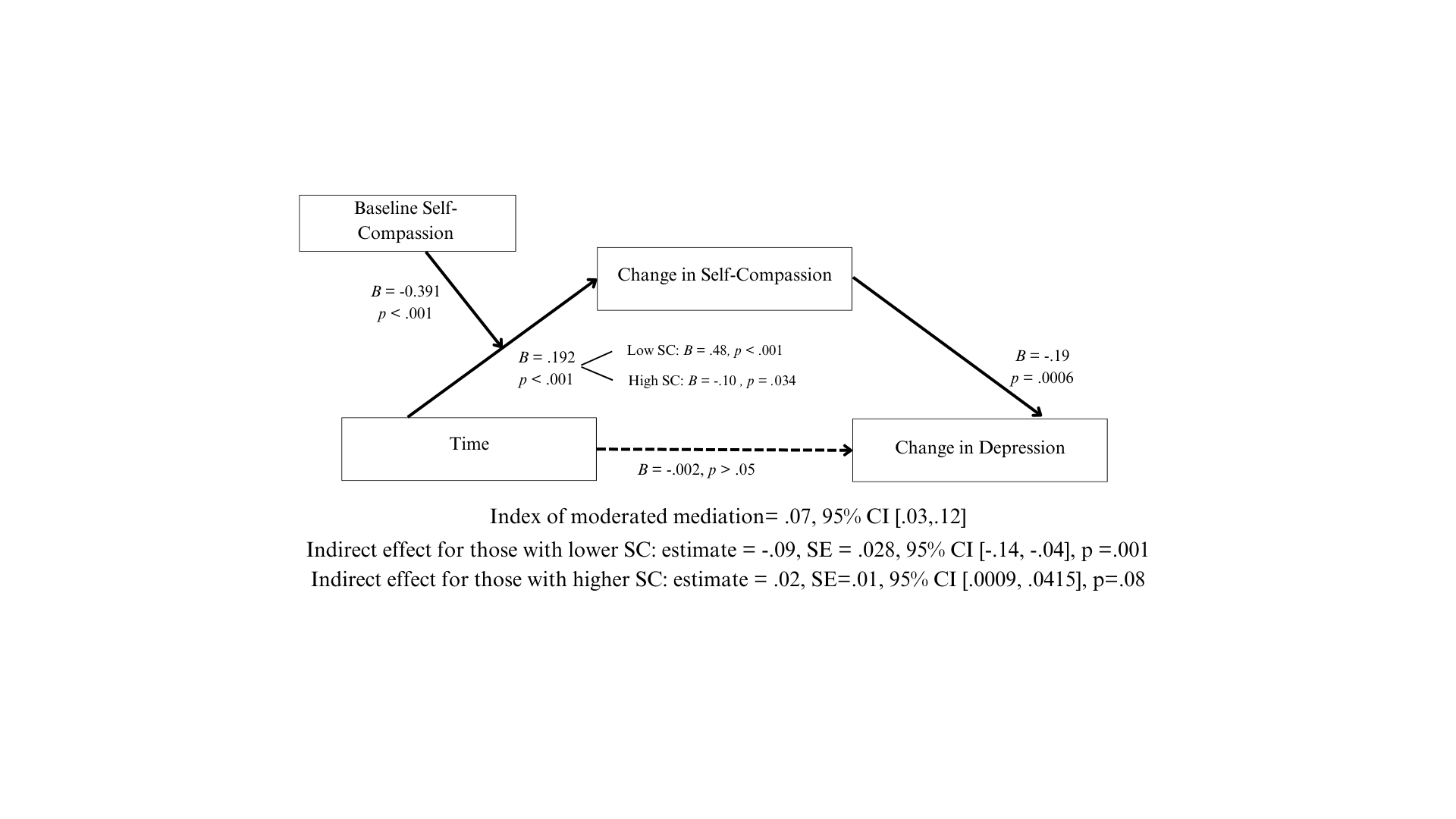


Index of moderated mediation = *estimate =* .07, 95% CI [.03,.12]

Indirect effect for those with lower baseline SC: *estimat*e = -.09, *SE* = .03, 95% CI [-.14, -.04], *p* =.001

Indirect effect for those with higher baseline SC: *estimate* = .02, *SE* = .01, 95% CI [.001, .04], *p* =.08

*Note*. SC = self-compassion.

**Online Resource 7**

*Estimated marginal means for depression at pre-intervention, post-intervention, and 18-month follow-up depending on baseline self-compassion.*

*M*_diff_ = 0.11, *p* = 0.02

*M*_diff_ = 0.12, *p* = 0.004

*Note*. SC = self-compassion.
